# Recollections of My Life

**Published:** 1900-12

**Authors:** 


### Reviews of Books.

**Recollections of my Life.** By Surgeon-General Sir JOSEPH FAYRER, Bart., K.C.S.I., LL.D., M.D., F.R.S., Q.H.P., etc. Pp. xii., 508. Edinburgh and London: William Blackwood and Sons. 1900.

There is a peculiar charm in reminiscences written by distinguished men, of large and varied experiences, who have always kept in touch with those in prominent positions, and have had social intercourse with people from all parts of the world and of various nationalities. A powerful influence for life is produced by early association with the sea and visits to distant countries, whereby fresh charms of scenery, and of animal and vegetable life, differences of dress, habits, and manners of the inhabitants, keep the mind in an incessant state of observation.

No men can have more, and few men as many, opportunities in a deep and intimate manner of gaining such accumulations of knowledge as medical men, while their early scientific training enables many of them to fill with skill and distinction positions of high importance quite outside the domain of their profession, even while practising it with great proficiency, and to write articles, books, and discourses of extreme value, increasing our knowledge at home of the peculiar maladies, climates, natural history, zoology, geology, and fauna of all parts of the world. It is deserving of special attention to notice what providential deliverances from imminent danger have frequently been granted to those who are destined in this manner to be blessings to their nation and fellow-creatures. All these points come into prominence in the perusal of this most remarkable, interesting, and highly instructive work which is now before us; giving to elder men greater knowledge than they possessed before of events in our history, such as the Indian Mutiny, the particulars of which thrilled them with horror, and the happy termination with profound gratitude, at the time of their occurrence.

Early life is well depicted, when sport, adventures and escapes from danger make strong impressions on the mind. An experience of medical interest is well described, where, when swimming in the sea off Bermuda, shortly after having seen a poor boy bitten in half at his waist and the lower half of his body swallowed by a shark, the author felt one of his legs tightly grasped, and, though he nearly sank with horror, he bravely struck out and reached a boat and was lifted into her, and then found that the long string of his drawers had wound round his leg and grasped it! The Indian Mutiny, with descriptions of the country, native princes, people and events which culminated in that awful episode of our history in that vast Empire, occupies a considerable space in the Recollections. It needs a brave man to recall, live again in, and describe those times of dread suspense, terror and treachery, and all the brutal massacres: but in contrast he is able to depict the calm waiting bravery of refined and delicate ladies, tender children, and noble men, and the electrifying ecstasy of the relief of Lucknow and Cawnpore, and the pure devotion of many faithful native servants. On all this the book must speak for itself. How touching are the words recorded of Sir Henry Lawrence, who, when he knew that his earth-life was near its close, requested that the only epitaph upon his tomb should be, "Here lies Henry Lawrence, who tried to do his duty." Of him and several other noble men excellent likenesses are inserted, including one of Sir Henry Durand, who began his grand and useful career by blowing up the gates of Ghuznee in the face of crowds of the enemy firing from the walls of the city: whose beautiful wife, accompanied by her faithful ayah, hiding by day and creeping by night, eventually reached a place of safety but died from the exhaustion induced by what she had passed through; and who has left two honoured sons, both knighted for distinguished service. The well-illustrated records of the Prince of Wales' visit to India, Sir Joseph's return to London, the honours, grandly earned, which were heaped upon him by his appreciative country, the International Medical Congress of 1881, the positions which he has been called upon to occupy, and other matters command attention in the careful perusal of a work of most profound interest and value; ending in the comforting knowledge that the talented author is still among us and has favoured us with an admirably finished likeness of himself,—a picture of power and modesty, which seems to say: "Not unto us, O Lord, not unto us, but unto Thy Name give the glory."

